# Effects of stellate ganglion block on perimenopausal hot flashes: a randomized controlled trial

**DOI:** 10.3389/fendo.2023.1293358

**Published:** 2023-11-28

**Authors:** Ying Li, Jia Chang, Gaoxiang Shi, Wenjing Zhang, Hui Wang, Lingyun Wei, Xiaochun Liu, Weiwei Zhang

**Affiliations:** ^1^ Third Hospital of Shanxi Medical University, Shanxi Bethune Hospital, Shanxi Academy of Medical Sciences, Tongji Shanxi Hospital, Taiyuan, China; ^2^ School of Nursing, Shanxi Medical University, Taiyuan, China

**Keywords:** stellate ganglion block, perimenopause, hot flashes, sleep quality, Kupperman Index and Pittsburgh Sleep Quality Index

## Abstract

**Background:**

Hot flashes are common symptoms afflicting perimenopausal women. A stellate ganglion block (SGB) is believed to be an effective treatment for hot flashes; however, more evidence is needed to evaluate its safety and efficacy in relieving perimenopausal hot flashes.

**Objective:**

To investigate the efficacy and safety of SGB for the treatment of perimenopausal hot flashes.

**Methods:**

A randomized controlled trial was conducted at Shanxi Bethune Hospital. Forty perimenopausal women with hot flashes were recruited from April 2022 to November 2022 and randomly assigned to receive either 6 consecutive SGB treatments or 6 consecutive saline placebo treatments. The primary outcome was the change in hot flash symptom score from baseline to 12 weeks after treatment. The secondary outcomes were the change in hot flash symptom score from baseline to 12 weeks after treatment and the post-treatment Kupperman Index (KI) and Pittsburgh Sleep Quality Index (PSQI) scores.

**Results:**

Of the 40 randomized subjects, 35 completed the study. All the variables were significantly improved. During 12 weeks of follow-up, the hot flash scores, Kupperman Menopause Scale scores, and Pittsburgh Sleep Quality Scale scores decreased significantly. Two subjects in the SGB treatment group experienced transient hoarseness, and the incidence of related adverse events was 10%. No related adverse events occurred in the control group.

**Conclusion:**

Compared to the control treatment, SGB treatment was a safe and effective nonhormone replacement therapy that significantly relieved perimenopausal hot flashes and effectively improved sleep quality. Additional studies are needed to assess the long-term efficacy of this therapy.

## 1 Introduction

Hot flashes, the most common symptoms of perimenopause, are characterized by sudden fluctuations in body temperature, as well as redness and profuse sweating of the face and neck, sometimes accompanied by chills (1). Although vasomotor disturbances, such as hot flashes, are not an organic impairment, severe hot flashes can significantly impact quality of life by increasing the risk of disrupted sleep, depression and anxiety, cognitive changes, and other serious illnesses. The incidence of hot flashes increases significantly during menopause, peaking in late perimenopause or early menopause, with approximately 80% of postmenopausal women experiencing hot flashes (2, 3). It has been reported that the incidence of hot flashes in sexually mature, perimenopausal, and menopausal women is as high as 21%, 30%, and 36%, respectively (4). It is extremely important to identify effective treatments to relieve the symptoms or reduce the incidence of hot flashes in perimenopausal and postmenopausal women.

Hormone replacement therapy is by far the most effective treatment for hot flashes(5), with an efficiency rate of 80-90% (6), but the frequency of replacement therapy is greatly limited by complications, such as headaches, premenstrual irritability and vaginal bleeding (7, 8), and contraindications, such as high risks of breast cancer, chronic heart disease, stroke or venous thromboembolism (9), and the possible risk of cancer due to hormones.

Some nonhormone replacement therapies or drug therapies are currently available. Paroxetine, a selective 5-hydroxytryptamine reuptake inhibitor (SSRI), is an effective and safe drug that has been approved for the relief of hot flashes as it significantly reduces hot flash scores despite being less effective than hormone replacement therapy (10). Venlafaxine, a selective norepinephrine reuptake inhibitor (SNRI), has also been shown to improve vasomotor symptoms, but there have been reports of interruption of treatment due to complications such as acute anemia (11). In addition, the effects of more moderate drugs, such as phytoestrogens and vitamin E, on hot flashes have been studied, and the results have been less than satisfactory^12^. It is necessary to look for a more effective and safer nonhormone replacement therapy to manage hot flashes in perimenopausal women.

A stellate ganglion block (SGB) is a well-established technique that is widely used to relieve sympathetic-mediated pain and improve vasomotor dysfunction by blocking the sympathetic ganglia in the lower cervical and upper thoracic spine with local anesthetic agents (12). Due to equipment improvements and technological innovations, SGB technology has evolved from blind puncture to precise puncture under guidance of X-rays and ultrasound. Ultrasound during SGB displays important anatomical structures such as blood vessels, thus greatly reducing the possibility of causing damage to important structures such as blood vessels, and significantly improving the safety of the procedure (13).

The exact mechanism by which SGB relieves hot flashes has not yet been clarified. It is hypothesized that SGB may relieve hot flashes by temporarily blocking the central temperature regulation mechanism (14). The effectiveness of SGB for hot flashes has been explored in breast cancer survivors. The results of previous experimental studies have shown good efficacy of SGB for hot flashes with relatively low rates of complications (15). The aim of our current study was to evaluate the safety and efficacy of SGB for the treatment of hot flashes in perimenopausal women through a prospective randomized controlled trial.

## 2 Methods

### 2.1 Participant

This was a single-blind randomized controlled trial with a follow-up period of three months that was conducted in Shanxi Bethune Hospital. Forty women who were aged 48-52 years with a gynecologic diagnosis of perimenopausal hot flashes were included in this study. The women had regular menstruation in the past. Women with acute infection, cardiorespiratory dysfunction, hepatic and renal insufficiency, neuropsychiatric disease, communication difficulties, coagulation disorders, who had undergone anticoagulant therapy, or were unable to be followed up were excluded from the study. The clinical trial protocol of this study was reviewed and approved by the Medical Ethics Committee of Shanxi Bethune Hospital (YXLL-2021-083), and registered in the Chinese Clinical Trials Registry (ChiCTR2300070017). All subjects signed the “Informed Consent” form before participating in this study, and all injections were performed by the same experienced anesthesiologist.

### 2.2 Randomization and blinding

The random number table method was used to achieve random grouping. Forty subjects were numbered from 1 to 40 in the order of their visit. Starting from any number in the random number table, a random number was assigned to each subject in turn. The 40 random numbers were arranged from small to large; odd random numbers were included in the experimental group, and even random numbers were included in the control group.

Double-blinding was impossible for this study because the presence of prominent Horner’s syndrome (ptosis, miosis, conjunctival hyperemia facial anhidrosis) on the same side was a sign of successful SGB block. Therefore, this was a single-blind trial, that is, the subjects were not informed of their grouping.

### 2.3 Operating procedure

**Figure 1:**
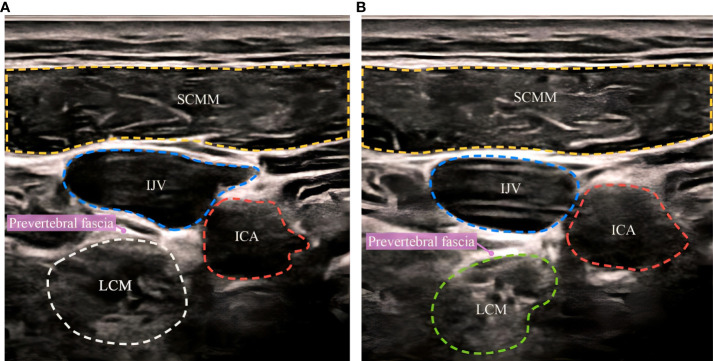
Ultrasound-guided stellate ganglion block before and after. **(A)** before SGB, **(B)** after SGB. SCMM, sternocleidomastoid muscle; IJV, internal jugular vein; ICA, internal carotid artery; LCM, longus collimuscle.

SCMM, sternocleidomastoid muscle; IJV, internal jugular vein; ICA, internal carotid artery; LCM, longus collimuscle.

The SGB procedures were performed under ultrasound guidance, and the operation was as follows. The patients were placed in the supine position, with their head turned to one side. A GE-LOGIQ5 ultrasonic diagnostic instrument was used for scanning, and the frequency of the ultrasonic probe was set to 10 MHz. Ultrasonic imaging was performed on the axial plane at the level of the cricoid cartilage, and the internal carotid artery and internal jugular vein were carefully distinguished. The stellate ganglion was located on the surface of the longus carotid muscle below the oblique internal carotid artery, and the 6th cervical vertebral body and the pretransverse tubercle stellate ganglion were punctured to a depth of 3.0-3.5 cm under ultrasound guidance, ensuring that no blood, cerebrospinal fluid or gas leaked, and 5 ml of 0.5% ropivacaine was infused slowly (**Figure 1A**). The needle tip was adjusted appropriately during the injection process so that the liquid could fully infiltrate the internal carotid artery, the cervical transverse process, and the entire stellate ganglion tissue. After the injection was completed (**Figure 1B**), the puncture point was compressed to stop bleeding for 5 minutes and the patient was changed to an upright sitting position. The anesthesiologist evaluated the patient for Horner syndrome and other adverse effects within 30 minutes (16). SGB treatment was performed once a day, alternating between the left and right side, for a total of 6 times. The saline control group was treated by the same operator in the same way, and 5 ml of 0.9% saline was used for the injection. In each group, the anesthesia machine, monitor, auxiliary ventilation equipment and other rescue equipment were in a standby state and equipped with necessary first aid drugs. The patients’ vital signs were monitored throughout the operation, and whether there was any abnormality in the patients’ consciousness or breathing were assessed. After the operation, the subjects were called back every week to ask whether there were any adverse reactions, and the subjects were reminded to follow up at the hospital on time.

### 2.4 Observed indicators

Hot flashes were quantified using a hot flash score for patients at baseline (one week before operation) and during follow-up. The hot flash score was obtained by multiplying the severity of hot flashes and the frequency of daily hot flashes (17). The severity of hot flashes was assessed using the scale reported in the Gwen Finck study, and the severity of hot flashes was divided into five grades (none= 0, mild=1, moderate=2, severe=3, very severe=4) (18).

Menopausal symptoms and sleep quality were quantified at baseline and during follow-up using the Kupperman Self-Rating Scale for Female Menopause and the Pittsburgh Sleep Quality Scale for further evaluation and analysis. The Kupperman self-rating scale is used to assess common symptoms of menopause, such as hot flashes, sweating, insomnia, etc., and assigns different weighting coefficients and adds them one by one to obtain the final Kupperman score (perimenopausal syndrome: mild: 15-20, moderate: 20-35, severe: > 35) (19). The Pittsburgh Sleep Quality Scale consists of 19 self-rated items grouped into 7 categories: subjective sleep quality, sleep latency, sleep duration, habitual sleep efficiency, cumulative sleep disturbance problems, sleep medication use, and daytime dysfunction. The scores from each category are added to obtain the final score (0-5: good sleep quality; 6-10: OK sleep quality; 11-15: average sleep quality; 16-20: poor sleep quality) (20). Lower scores indicate better relief of hot flashes and menopausal symptoms and improved sleep quality.

### 2.5 Statistical analysis

**Figure 2:**
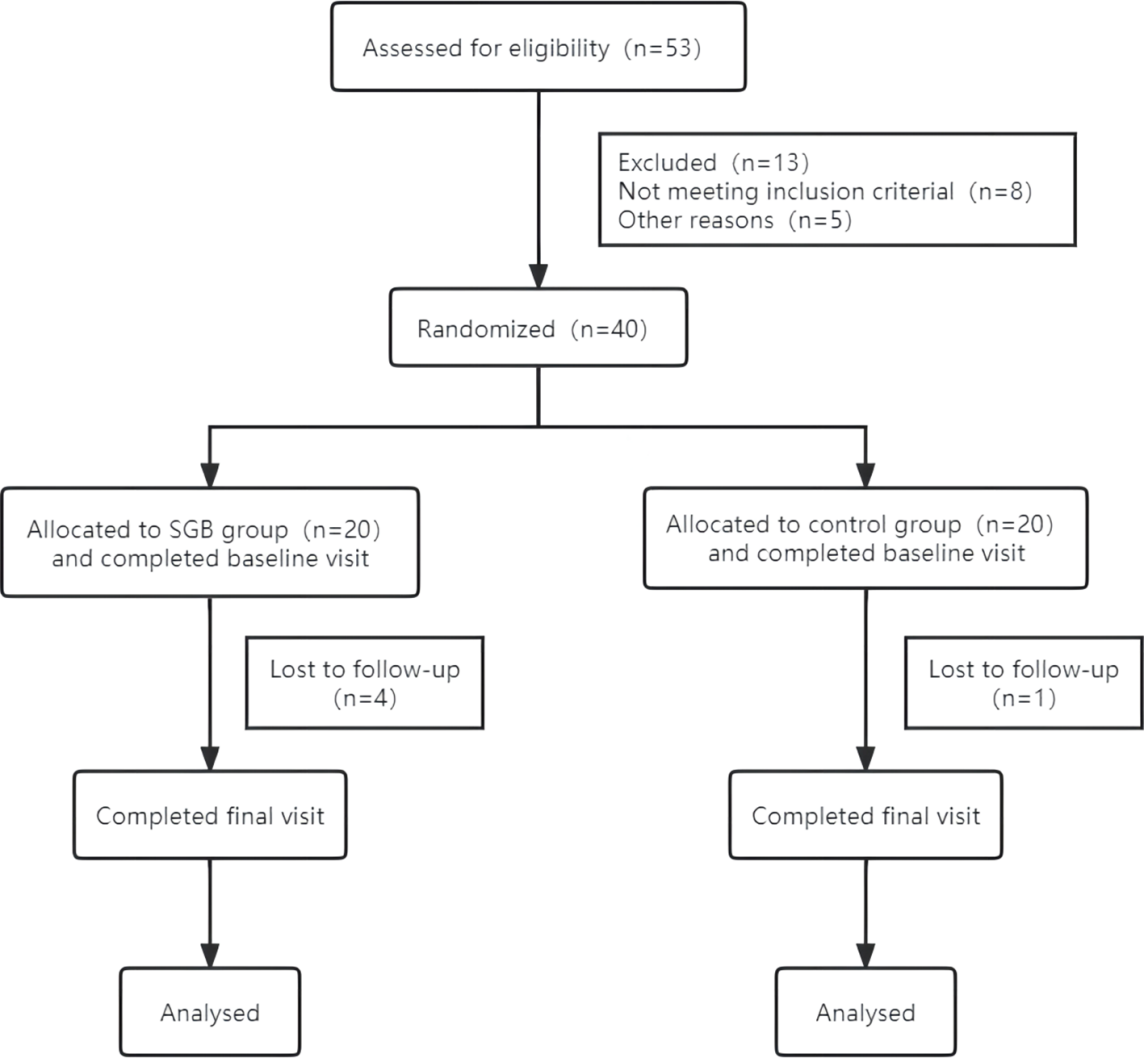
Flowchart for participant selection.

The sample size was estimated based on data from previous studies in the literature (21), assuming a one-sided significance level of 0.05 and a test efficacy of 90%, and 20 cases per group were required with an estimated 20% loss to follow-up. The flow chart of this study is shown in **Figure 2**.

Quantitative data are described as the mean ± standard deviation. For those with a normal distribution, one-way ANOVA was used to analyze the differences in each indicator at the four specified time points. The data of the two groups at the 4 specified time points were compared between the groups. Homogenous and unequal variances were analyzed with t tests. Those nonnormally distributed were tested by the Wilcoxon rank sum test. The chi-square test was used to compare the differences in the incidence of adverse events between the two groups.

IBM SPSS 26.0 was used for statistical analysis, and a P value < 0.05 was considered statistically significant.

## 3 Results

### 3.1 General characteristics

Forty women with a gynaecologic diagnosis of perimenopausal hot flashes underwent a three-month intervention and follow-up (**Figure 2**); 20 were included in the SGB treatment group and 20 were included in the saline control group. During the follow-up period, five patients were lost to follow-up, four of whom were in the SGB group (one patient was not followed up due to postoperative hoarseness, two patients did not participate in follow-up beyond 8 weeks due to unknown reasons, and one patient did not undergo the operation due to the impact of the COVID-19 pandemic). There was one case in the control group (the follow-up at the 12th week after the operation was not performed due to ineffective treatment).

**Table 1** shows the population and basic clinical characteristics of the SGB group and control group, and no statistically significant differences were observed between the two groups. There was no significant difference in hot flash score, hot flash frequency, Kupperman score, or Pittsburgh sleep quality score between the two groups before the intervention (at baseline), and they were comparable.

**Table 1:** Demographic and clinical characteristics of the treatment group at baseline.

	treatment group		P
	SGB(n=20)	control(n=20)	
age(y)	49.60 ± 1.43	49.75 ± 1.37	0.737
BMI(kg/m2)	23.47 ± 2.02	22.58 ± 1.67	0.134
Menstrual disorder how long(m)	5.65 ± 1.46	5.85 ± 1.42	0.664
KI score	28.90 ± 8.71	26.85 ± 12.64	0.554
PSQI score	10.80 ± 1.89	11.20 ± 2.82	0.601
hot flash score	15.55 ± 14.72	15.15 ± 5.37	0.910
diary flush frequency	10.15 ± 6.80	10.55 ± 3.44	0.816

The general data of the subjects in the two groups are presented as the mean ± standard deviation. SGB, stellate ganglion block; BMI, body mass index; KI, Kupperman index; PSQI, Pittsburgh Sleep Quality Index. There were no statistically significant differences in the general data between the two groups.

### 3.2 Main results

The subjects’ hot flash scores and hot flash frequency data are shown in **Table 2**. At baseline, the mean hot flash score in the SGB group was 15.55 (95% CI, 8.66-22.44), of which 55% were mild and 45% were moderate. The mean hot flash score in the saline group was 15.15 (95% CI, 12.64-17.66), with 50% being mild and 50% being moderate. The statistical analysis of hot flash score and hot flash frequency showed that in the 12th week, hot flash symptoms were significantly relieved in the SGB group, with an average difference of 13.92 and 8.52, respectively. The difference was statistically significant. The control group showed no statistically significant change from baseline.

**Table 2:** Comparison of hot flash indicators between the two groups of subjects at different time points.

		baseline	4 weeks	8 weeks	12 weeks
		*n* = 20	*n*_SGB_ = 16, *n*_cont_ = 19		
hot flash score	SGB	15.55 ± 14.72	5.45 ± 3.55^a^	3.85 ± 2.60^b^	1.63 ± 0.74^c^
	control	15.15 ± 5.37	13.95 ± 4.39^a^*	15.00 ± 5.76^b^*	15.30 ± 5.48^c^*
	*P* value	0.910	<0.001	<0.001	<0.001
diary flush frequency	SGB	10.15 ± 6.80	5.45 ± 3.55^a^	3.85 ± 2.60^b^	1.63 ± 0.74^c^
	control	10.55 ± 3.44	9.70 ± 2.56^a^*	10.25 ± 2.88^b^*	10.70 ± 3.54^c^*
	*P* value	0.816	<0.001	<0.001	<0.001
Hot flash severity	SGB	1.45 ± 0.51	1.00 ± 0.00^a^	0.80 ± 0.41^b^	0.63 ± 0.49^c^
	control	1.50 ± 0.51	1.50 ± 0.51^a^*	1.50 ± 0.51^b^*	1.50 ± 0.51^c^*
	*P* value	0.759	<0.001	<0.001	<0.001

a compared to the baseline, P<0.05.

b compared to the baseline, P<0.05.

c compared to the baseline, P<0.05.

^*^compared to the SGB group, P<0.001.

**Figure 3:**
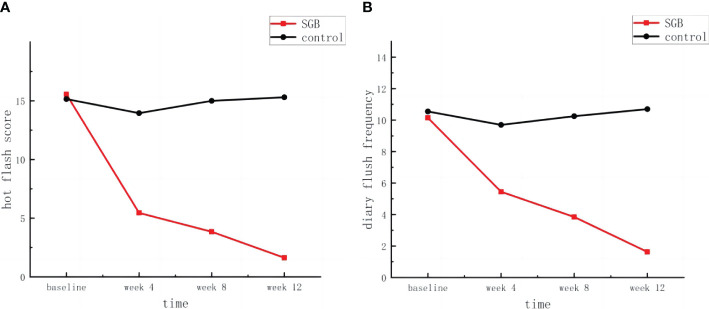
**(A)** Hot flash score and, **(B)** diary flush frequency.

As shown in **Figure 3**, the SGB group had the greatest relief of hot flashes during the first 4 weeks of follow-up, with a reduction of 10.1 and 4.7 in hot flash score and hot flash frequency, respectively, when compared with the baseline.

Data points represent the mean value for each time point.

### 3.3 Secondary results

**Figure 4:**
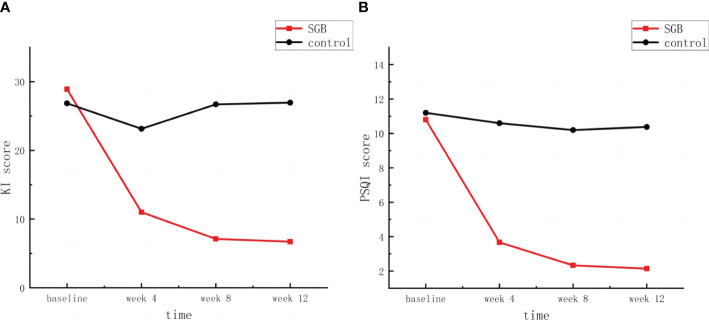
**(A)** Kupperman index score and **(B)** Pittsburgh sleep quality index score.

As shown in **Figure 4**, compared with that in the control group, the Kupperman Menopause Scale score in the SGB group was more greatly decreased from the baseline. The mean decrease was 17.90 in the 4th week, 21.79 in the 8th week, and 22.15 in the 12th week. The Kupperman scores during the follow-up period were significantly different in the between-group analysis (**Table 3**).

**Table 3:** Comparison of the Kupperman index score and Pittsburgh sleep quality index score between the two groups of subjects at different time points.

		baseline	4 weeks	8 weeks	12 weeks
		*n* = 20	*n*_SGB_ = 16, *n*_cont_ = 19		
KI score	SGB	28.90 ± 8.71	11.00 ± 4.06^a^	7.11 ± 2.93^b^	6.71 ± 3.63^c^
	control	26.85 ± 12.64	23.15 ± 9.79^a^*	26.70 ± 12.01^b^*	26.95 ± 13.19^c^*
	*P* value	0.554	<0.001	<0.001	<0.001
PSQI score	SGB	10.80 ± 1.88	3.67 ± 1.53a	2.33 ± 1.61b	2.14 ± 1.87c
	control	11.20 ± 2.82	10.60 ± 2.60^a^*	10.20 ± 2.14^b^*	10.38 ± 2.58^c^*
	*P* value	0.601	<0.001	<0.001	<0.001

KI, Kupperman index; PSQI, Pittsburgh Sleep Quality Index.

a compared to the baseline, P<0.05.

b compared to the baseline, P<0.05.

c compared to the baseline, P<0.05.

^*^compared to the SGB group, P<0.001.

Data points represent the mean value for each time point.

Changes in Pittsburgh Sleep Scale scores from baseline to the 12th week were similar to changes in the Kupperman Menopause Scale scores.

### 3.4 Safety evaluation

Only 2 participants in the SGB group had transient hoarseness, a treatment-related adverse event, that did not recur during the follow-up period. No adverse events unrelated to treatment occurred. Participants in the control group did not report any adverse reactions (**Table 4**).

**Table 4:** The incidence of adverse events in the two groups.

	hoarseness	dizziness	cough	upper limb numbness	pneumothorax	infections at the puncture site	haematoma at the puncture site	total
SGB	2(10%)	0(0)	0(0)	0(0)	0(0)	0(0)	0(0)	2(10%)
Control	0(0)	0(0)	0(0)	0(0)	0(0)	0(0)	0(0)	0(0)
χ^2^ value	0.526							
*P* value	0.468							

There were no statistically significant differences in the safety evaluation between the two groups.

## 4 Discussion

SGB is widely used in the treatment of chronic pain and complex regional localized pain syndrome (22–24) as it improves the prognosis of drug-refractory ventricular arrhythmias (25) and posttraumatic stress disorders (PTSD) (26). Currently, the impact of SGB on vasodilatory symptoms, such as hot flashes and night sweats, has been studied in depth (27).

The clinical data related to the effect of SGB on hot flash treatment are limited, and most are case reports, so the level of evidence is not high (28). Due to its invasive nature and lack of evidence from large, long-term randomized controlled trials, the North American Menopause Society classified SGB as a cautionary recommendation, and more trials are needed to demonstrate its safety and efficacy^12^. This is the first randomized controlled trial to study the effect of SGB on hot flashes in normal perimenopausal women.

SGB is considered a relatively safe clinical procedure, with a serious adverse reaction rate of 1.7/1000 as reported by Wulf and Maier (29). Most complications are temporary and mostly found during or shortly after the procedure; the most common adverse events are hoarseness and dizziness (30). During the three-month follow-up of the subjects in this study, only 2 showed temporary hoarseness within a few hours after SGB, but no adverse reactions recurred during the follow-up period. In addition, there were no reports of serious adverse reactions or events unrelated to treatment. This study provides additional clinical evidence for the safety of SGB.

The mechanism of SGB in preventing and treating hot flashes has not yet been elucidated as it is a block of sympathetic nerve conduction. Lipov believes that the therapeutic effect is based on interrupting the connection between the central nervous system and the sympathetic nervous system (31). Freedman proposed that the thermoneutral zone is narrowed, that the caudate nucleus may be stimulated by environmental stress in a state of sympathetic excitation and that SGB can reduce the stress of sympathetic stimulation and restore the normal state (5). In addition, the therapeutic effect of SGB may also involve the reduction of nerve growth factor levels (32).

SGB was initially used in the treatment of hyperhidrosis, which has similarities to hot flashes in terms of symptom presentation (33). Walega’s study showed that SGB treatment can significantly reduce vasomotor symptoms in postmenopausal women (34). In our study, improvement in hot flash symptoms was observed according to the hot flash score, similar to the results of a previous study (15). Consistent with the results of Walega’s study, subjects treated with SGB had significantly less severe and less frequent hot flashes over the 12-week follow-up period, with a 13.92 reduction in hot flash scores and an 8.52 reduction in hot flash frequency after SGB treatment. Additionally, we found that the effect was most pronounced in the first 4 weeks after treatment, which is consistent with the findings reported by Haest (15). However, Rahimzadeh found that the greatest degree of remission was observed at 2 weeks postoperatively, and we were unable perform a comparison with this result due to the relatively long follow-up interval (35). However, Othman’s study showed a statistically significant increase in hot flash frequency at 4 weeks postoperatively (36). This suggests that additional randomized controlled trials are needed to verify changes in the frequency of hot flashes after 4 weeks of treatment. The hot flash scores were further decreased at the two-month follow-up, but the downwards trend gradually slowed. Our findings support the feasibility of SGB for the treatment of hot flashes in perimenopausal women.

Sleep disturbance is another major concern for perimenopausal women and is often accompanied by hot flashes (37). In the current study, sleep quality improved as the severity and frequency of hot flashes were reduced. That is, there was a significant improvement in the first 4 weeks, and the improvement in sleep quality was relatively slight after 4 weeks. Similarly, other perimenopausal symptoms (such as irritability, dyspareunia, dizziness, fatigue, etc.) were significantly improved along with the improvement of hot flashes and sleep quality.

This study has certain limitations. First, the patients who underwent SGB treatment exhibited significant Horner syndrome, which prevented double-blinding between the investigator and the study subjects and caused possible subjective information bias of the investigator. Second, the sample size of this study was relatively small, not a multicentre, large-sample clinical study with good representation, and PP analysis may have caused overestimation of the treatment effect and reduced the reliability of the experimental results. Third, the goal of this trial is to seek a long-term effective method to alleviate hot flashes in perimenopausal or even postmenopausal women, and the follow-up period of this trial is far from adequate due to research funding constraints and other reasons. Fourth, the time interval between the subjects’ visits to the hospital and the evaluation was too long, and our trial indicators, including hot flash score, hot flash frequency, Kupperman scale, and Pittsburgh sleep scale, originated from subjects’ subjective evaluation. Fifth, in our trial, we only compared the efficacy of SGB with that of saline and did not compare the difference between hormone replacement therapy, paroxetine, or any other pharmacological treatments. A comparison of the safety and efficacy of several therapies would have revealed the best recommendation for hot flash relief.

## 5 Conclusion

A SGB was safe and effective for relieving hot flashes and improving sleep quality in perimenopausal women in a 3-month study. Larger sample sizes, longer follow-up times, and more frequent follow-up are needed to further understand the long-term efficacy and mechanism of action of this treatment modality.

## Data availability statement

The raw data supporting the conclusions of this article will be made available by the authors, without undue reservation.

## Ethics statement

The studies involving humans were approved by Medical Ethics Committee, Shanxi Academy of Medical Sciences, Shanxi Bethune Hospital. The studies were conducted in accordance with the local legislation and institutional requirements. The participants provided their written informed consent to participate in this study. Written informed consent was obtained from the individual(s) for the publication of any potentially identifiable images or data included in this article.

## Author contributions

YL: Writing – original draft. JC: Writing – original draft. GS: Writing – review & editing. WJZ: Writing – review & editing. HW: Writing – review & editing. LW: Writing – review & editing. XL: Writing – review & editing. WWZ: Writing – review & editing.
